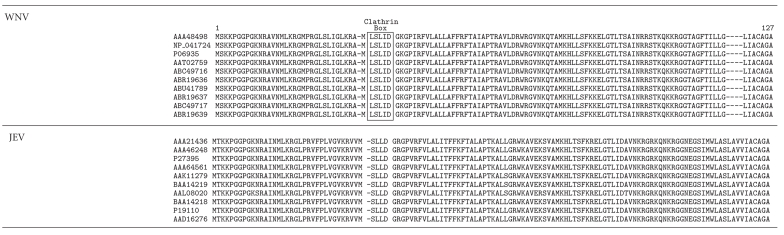# Evolution and Diversity of the Human Hepatitis D Virus Genome

**DOI:** 10.1155/2010/323654

**Published:** 2010-02-24

**Authors:** Chi-Ruei Huang, Szecheng J. Lo

**Affiliations:** ^1^Institute of Microbiology and Immunology, National Yang Ming University, Taipei 112, Taiwan; ^2^Department of Life Science, Chang Gung University, TaoYuan 333, Taiwan

## Abstract

Human hepatitis delta virus (HDV) is the smallest RNA virus in genome. HDV genome is divided into a viroid-like sequence and a protein-coding sequence which could have originated from different resources and the HDV genome was eventually constituted through RNA recombination. The genome subsequently diversified through accumulation of mutations selected by interactions between the mutated RNA and proteins with host factors to successfully form the infectious virions. Therefore, we propose that the conservation of HDV nucleotide sequence is highly related with its functionality. Genome analysis of known HDV isolates shows that the C-terminal coding sequences of large delta antigen (LDAg) are the highest diversity than other regions of protein-coding sequences but they still retain biological functionality to interact with the heavy chain of clathrin can be selected and maintained. Since viruses interact with many host factors, including escaping the host immune response, how to design a program to predict RNA genome evolution is a great challenging work.

## 1. Introduction

Viruses are aheterogeneous class of agents that parasitize every form of life including not only animals and plants but also bacteria, archaea, and fungi. Viruses vary greatly in particle size and morphology and in genetic complexity and host range. There are the DNA and the RNA viruses defined by the type of nucleic acid present in the mature virion particles. The genome size of the virus, be it a DNA or RNA virus, determines the number of proteins encoded by the viral genome. The minimal set of viral proteins includes capsid and envelope structural proteins required for the assembly of the virion particles and DNA or RNA polymerase for replication of the viral genome. A subclass of viruses, containing only few members, has been called satellite viruses since their genome can be encapsidated by their own coat proteins but require helper viruses for providing envelopes for assembling mature virions.

The length of the genome of a DNA virus varies from a few kilo-bases (kb) to several hundred kb. The smallest known DNA virus is the human hepatitis B virus (HBV) which is 3.2 kb long and contains four open reading frames (ORFs) that encode the surface antigens, the core proteins, a polymerase, and an X protein [1, 2]. Together with the duck HBV (DHBV), the woodchuck hepatitis virus (WHV), and the ground squirrel hepatitis virus (GSHV), they form a family of DNA viruses called the hepadnaviruses [3]. The genome size of RNA viruses is generally shorter than that of DNA viruses and ranges approximately from 2 to 31 kb. The smallest RNA virus identified to date is the human hepatitis D virus (HDV) which is about 1.7 kb in size and contains only one ORF [4–7]. HDV requires the coexistence of HBV to supply envelope proteins for its assembly into mature virions and is, hence, a defective virus, or it is called a satellite virus of HBV [4, 6]. Although HBV and other hepadnaviruses are found in a number of mammals, HDV has thus far been found in humans [8]. There is another unique class of RNA-containing infectious agents called the viroid. Viroid RNA is a circular genome with a few hundred nucleotides long and carries no discernible coding sequences. Unlike other RNA viruses, viroids exist as a naked form of RNA without a capsid or coat-protein armor. Viroids are known to infect many species of plants and have not been found in any other life forms besides plants [9]. 

The origin of viruses remains elusive and debatable because there are no fossils of viruses. Currently, there are two popular hypotheses to explain possible origin of viruses, viz the “regressive” or “degeneracy hypothesis” and the “cellular origin” or “vagrancy hypothesis” [10, 11]. The “regressive hypothesis” is similar to the “endosymbiosis hypothesis” which explains the origin of mitochondria and chloroplasts. Both hypotheses propose that the two indispensable cellular organelles that harbor their own genetic content originated from small prokaryotic cells that came to reside within some ancestral eukaryotic cells and gradually degenerated to become organelles of specific biological functions. Therefore, vaccinia viruses, each of which harbors a DNA genome of about 200 kb in size, might have originated from parasitized smaller cells through successive reduction of the genome size within the host cells. The currently existing fact that, cell within cell, such as rickettsia and Chlamydia, can only replicate within the host cells supports the “regressive hypothesis.” 

The “cellular origin hypothesis” is based on the finding of the presence of plasmids and various types of mobile elements, such as transposons and retrotransposon, in the current prokaryotes and eukaryotes. When present in ancient cells, these elements could evolve to become viruses when they had gained new properties through evolutionary divergence to enable them to escape from the entrapping host cells to gain entry to and propagate in new hosts. Retroviruses and HBV, despite being different in the RNA or DNA content, respectively, might have originated from the same family of ancestral retrotransposons since replication of both viral genomes involves reverse transcription [12, 13]. Hence, the distinguishing feature between the “regressive” and the “cellular origin” hypotheses lies in the “loss” or “gain” of genetic materials, respectively. Nonetheless, the hypotheses are similar in the same prerequisite that viruses evolved subsequent to the emergence of self-sustaining cells on Earth. A third hypothesis, called the “coevolution hypothesis”, postulates that viruses had evolved from the ancient soup of complex protein and nucleic acid molecules independent of the self-sustaining host cells and concurrent in time point with the appearance of the first cells on Earth [14]. 

However, no single hypothesis can satisfactorily explain the origin all known viruses. In this paper, we use the smallest human RNA virus, HDV, to illustrate how this virus could have evolved and diverged. The evolution of larger genome size of RNA viruses, such as retroviruses, flaviruses, picornavirus, and corona viruses, is not a subject in this review.

## 2. Molecular Biology of HDV

HDV was first discovered in HBV-infected patients by an Italian physician, Mario Rizzetto, in 1977. It was originally thought to be a new nuclear antigen associated with HBV [15]. It was later proved to be a new virus that requires the surface antigens of HBV (HBsAgs) to support its life cycle and infectivity as clearly demonstrated in the experimental animals, chimpanzee, and woodchuck [8, 16, 17]. Co- or superinfection by HDV in HBV patients is closely correlated with the more severe symptoms of liver disease HBV infection alone [18, 19]. Studies have established that the HDV genome is a negative circular RNA about 1.7 kb long. Electron microscopic observation has further revealed that the HDV genome appears as a rod-shape structure under nondenaturing conditions but the genome presents itself in a circular form under denaturing conditions [20]. Extensive intramolecular base-pairing of the single-stranded RNA molecule results in the formation of the double-stranded rod-like structure [20].

**Figure 1:**
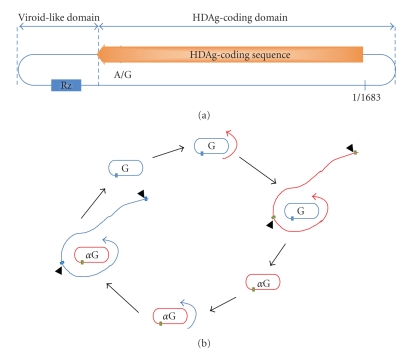
HDV genome and replication. (a) Structural features of the HDV genome. The viral genome is a single-stranded circular RNA molecule composing of the viroid-like and the HDAg-coding domains. The ribozyme sequence (Rz) in the viroid-like domain is shown as a blue box. “A/G” in the HDAg-coding sequence indicates the nucleotide edited by the host ADAR leading to the synthesis of LDAg. The beginning and the end of the circular genome are labeled as 1/1683. (b) Replication of the HDV genome by the double rolling-circle replication model. The genome and antigenome of HDV are represented by blue or red circles and labeled as “G” or “*α*G”, respectively. The open blue or red lines represent the primary transcription products composed of multimeric units of the viral genome or antigenome, respectively. The location of Rz is denoted by the green or blue boxes in the genome and antigenome, respectively. Arrowheads indicate the self-cleavage site of the ribozyme.

Most RNA viruses encode their own replicases or RNA-dependent RNA polymerases (RdRp) essential for viral genome replication. However, the HDV genome does not carry a replicase gene rendering HDV totally dependent on the host replication machinery for its propagation [21]. It has been demonstrated that HDV uses the host DNA-dependent RNA polymerases (DdRp) to facilitate the replication of its genome and antigenome [21] through a double-rolling circle mechanism (Figure 1) as the majority of viroids does [22]. Unlike viroid, however, HDV requires the HDV genome-encoded small delta antigen, SDAg, for replication. Furthermore, HDV is 4-5-fold larger in genome size than the viroid. In addition to the coding sequence, the HDV genome contains a viroid-like sequence in both the genome and the antigenome [23–25]. A ribozyme, a self-cleaving RNA sequence, resides in the viroid-like sequence of the HDV genome that cleaves a linear form of multiple-copy length of the viral genome or antigenome into monomeric units that are then circularized to complete the replication cycle (Figure 1). 

The HDV genome has only one ORF to encode two *isoforms* of hepatitis delta antigen (HDAg) during replication. The small hepatitis delta antigen, SDAg, is a 24-kDa protein composed of 195 amino acid residues; the large hepatitis delta antigen, LDAg, is 27 kDa and consists of 214 residues [26, 27]. The two antigens are identical at the N-terminal 195 residues. The extra 19 residues (or 20 residues in those HDV isolates from South America) present in the C-terminus of LDAg are derived from RNA editing catalyzed by the host enzyme, adenosine deaminase acting on the RNA (ADAR), and converting the stop codon (UAG) of the SDAg ORF into a tryptophan codon (UGG), thus extending the coding sequence to terminate at a downstream termination codon [28–34]. In the life cycle of HDV, SDAg supports viral genome replication while LDAg inhibits replication and promotes interaction of the HDV genome with HBsAgs for virion packaging [35–37]. In spite of the sharing of the same sequence of 195 amino acid residues between SDAg and LDAg, the additional peptide sequence of 19-20 residues of LDAg has a distinct biological function. The peptide sequence contains a nuclear export signal (NES) that facilitates nuclear exportation of HDAg and an isoprenylation recognition motif that interacts with HBsAgs to form the HDV virions [37–41].

## 3. Diversity of HDV Genome Sequences

Based on the percentage of nucleotide identity of the genome, HDV was initially classified into three genotypes, designated genotypes I-III [4]. The distribution of various HDV genotypes is closely associated with geographic origins and disease outcomes [18]. Genotype I HDV is distributed worldwide and causes hepatitis with a wide range of clinical severity. Genotype II HDV is found mainly in East and North Asia including Taiwan, Japan, and Siberia and infection usually leads to less severe clinical manifestations than genotype I infection. On the other hand, genotype III is reported to cause a severe form of fulminant hepatitis and has only been isolated in the northern area of South America. In addition, the nucleotide sequence of genotype III is the most divergent amongst all the isolated HDV sequences [42]. 

Recently, an increasing number of HDV isolates have been identified and sequenced. The classification of HDV thus changes from the three genotypes into the eight clades, HDV1 to HDV8 [42, 43]. In the new scheme of HDV classification, HDV1-3 has replaced genotypes I-III, respectively. HDV isolates that belong to genotype II subgroup b are now regrouped into the HDV4 class. The most recently isolated HDV sequences derived from African patients are grouped into HDV5 through to HDV8 [42, 43]. HDV sequences isolated from various geographic locations of Africa are found to be more divergent sequences isolated from other continents suggesting that the first HDV might have arisen from Africans. Two related questions are then raised: (1) How did the first HDV originate and how was it evolved? (2) Is HDV diversity correlated with the established waves of human migration across continents?

**Figure 2:**
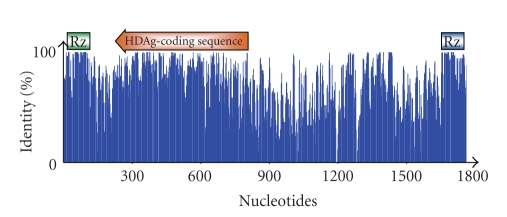
Profiling of nucleotide identities of 43 HDV isolates. The vertical-axis represents percentage nucleotide identity and the horizontal-axis displays the whole viral genome sequence as the relative length of 1800 nucleotides due to the alignment. The ribozyme (Rz) in the genome and antigenome is indicated by the green or blue box, respectively. The relative genomic position of the the HDAg-coding sequence is also shown (red arrow-bar).

Analysis of the nucleotide sequences of 43 HDV isolates mined from databases in the public domain has revealed that the percentage nucleotide sequence identity of the complete HDV genome ranges from 64.1% to 76.4% in comparison of each out-group [42]. The percentage identify is the lowest (64.1%) between HDV3 and HDV 5 and the highest (76.4%). However, the percentage identity of the SDAg coding sequence ranges from 72.4% to 83.6% in comparison of each out-group in which the lowest (72.4%) is found between HDV3 and HDV7 and the highest (83.6%) is between HDV2 and HDV5 [42]. A higher percentage identity of the SDAg coding sequence than that of the completeviral genomic sequence indicates that nucleotide sequence diversity is more restricted to the functional region than in the noncoding region. Profiling of nucleotide identities from the 43 HDV isolates shows that, when compared to the HDAg coding sequence, the more conserved nucleotide sequence is found in the viroid-like sequence that contains a ribozyme sequence (Figure 2). The reason for lower diversity in the viroid-like region than in the HDAg coding sequence could be because of the usage of wobble codons in HDAg translation contributing to a higher degree of tolerance of nucleotide substitutions without affecting the amino acid residue. On the other hand, the ribozyme in the viroid-like region exerts its function directly in the RNA sequence and is, hence, less tolerant to nucleotide changes. This proposition is supported by a base substitution study that revealed a 2.4-fold higher C-to-U substitution in the HDV coding sequence than in the full-length genome echoing frequent C-to-U codon degeneracy in the third base of codons [42].

## 4. The Origin of HDV

The current hypothesis of the origin of HDV favors the “cellular origin” concept because numerous studies have found similarities in structures and sequences between the nucleotide and protein sequences of cellular genes and the coding and viroid-like sequences of HDV. 

In a genomewide search for ribozyme sequences, it has been found that an HDV-like sequence in the human *CPEB3* gene encodes the cytoplasmic polyadenylation element-binding protein 3 [44]. CPEB3 is a member of a family of proteins that regulate mRNA polyadenylation and are highly conserved among mammals. The ribozyme resides in the second intron of *CPEB3* but is dissimilar in the primary nucleotide sequence to that of the HDV ribozyme. However, the secondary structure of the *CPEB3* ribozyme is similar to that of the HDV ribozyme both of which serve the function of self-cleavage of the multimeric precursor. These findings provide a clue that the viroid-like sequence of HDV could have arisen from an ancestral *CPEB3* ribozyme in the mammal. Two other HDV viroid-like sequences with sequence similarity to a cellular RNA come from the findings that the HDV genomic sequence from nucleotide (nt) 683–724 is complementary to nt 10–55 of the 7SL RNA sequence and the antigneomic sequence from nt 858–899 is complementary to nt 188–233 also of the 7SL RNA [45, 46]. Both complementary regions of the HDV sequence are located adjacent to the viroid-like region and have 73% to 77% nucleotide identity to the 7SL RNA [45]. 

A cellular protein, termed delta interacting protein A (DIPA), is reported to interact with HDAg affecting HDV replication [47]. Alignment of the protein sequences of DIPA and HDAg has revealed a sequence identity of 24% and sequence similarity of 56% [47]. Both DIPA and HDAg are similar in size and both form oligomers through a coiled-coil domain. These authors have further proposed that the *DIPA* gene is a homolog of HDV and that the capture of some *DIPA* transcripts by a viroid-like sequence could have initiated the evolution of the very first HDV. Findings of RNA recombination between different HDV genotypes in patients or in cell cultures support the idea that DIPA transcripts and viroid-like sequence could join together [41, 48, 49]. Two different groups of researchers have further demonstrated that RNA recombination is through a switch in the transcription templates [50, 51]. 

This “cellular origin hypothesis” then raises the interesting questions of when and in what animal host(s) such RNA recombination first occurred. Since HDV has to coexist with HBV for propagation, a first guess would be that HDV should have evolved in animals that were susceptible to infection by hepadnaviruses. The most primitive HBV is thought to DHBV found in ducks, the viral genome of which contains three ORFs but lacks the X-protein ORF that is found in the human HBV [2, 52]. However, no naturally occurring HDV has so far been found in ducks nor in two other mammals, woodchucks, and ground squirrels, despite the presence of the X-protein ORF in the corresponding hepadnaviruses. Taken together, it is highly likely that the first HDV originated in ancestral humans. Thereafter, HDV coevolved with HBV and selected human cellular factors to generate sequence diversity along with the many waves of human migration to different geographic localities.

## 5. HDV Sequence Diversity through Mutations Followed by Host Factors' Selection

Mutations frequently occur as a result of nucleotide mismatches during DNA or RNA replication. In general, the mutation rate of RNA viruses is higher than that of DNA viruses because the fidelity of base-pairing is lower during RNA replication than DNA replication. Diversity of the RNA genome of HDV should, therefore, be derived primarily from accumulation of mutations. Mutations that had occurred in essential sequences that could lead to impairment of virion formation could have been eliminated during viral replication. In contrast, mutations in nonessential sequences could have been preserved.

In addition to the ribozyme sequence in the viroid-like region and the HDAg coding sequence, there are several *cis*-elements important for HDV replication. An essential *cis*-element is the promoter sequence that is recognized by cellular DdRps for transcription. Another important *cis* sequence is recognized by ADARs for RNA editing to result in LDAg production. 

Among the essential sequences for HDV replication and maturation, the sequence encoding the C-terminal peptide of LDAg is highly variable among the HDV1-8; ^198^ILFPADPPFSPQSCCRPQ^214^ in HDV1 (as Group I), ^198^GPSPPQQRLPLLECTPQ^214^ in HDV2 and HDV4-8 (as Group II), and ^198^FTPPPPGYYWVPGCTQQ^214^ in HDV3 (as group III) (Table 1). The identityof the amino acid sequence of the in-groups is almost 100%; however, the nucleotide substitution of in-groups ranges from 0.42% to 7.31% (Table 1). There are three functional domains in this short peptide of LDAg. They are the nuclear exporting signal, the clathrin heavy chain (CHC) interacting domain, and the isoprenylation signal [39, 53–55]. There is no nucleotide substitution occurring in the clathrin box and isoprenylation motif within Group III, while 3.60% and 0.19% within Group I, and 0.77% and 4.5% within Group II (Table 1). The lower identity among the various clades could be attributed to the relaxation of the amino acid sequence participating in interaction with its counterparts of cellular proteins and the flexibility of cellular location. For example, the last four amino acids called the CaaX box (C: cysteine; a: aliphatic amino acids; X: any amino acid except leucine and phenylalanine) is an isoprenylation signal required for interaction with HBsAgs for virion maturation. The isoprenylation signal of HDV1 (Group I) is ^211^CRPQ^214^, HDV2 and HDV4 through to HDV8 (Group II) is ^211^CTPQ^214^, and HDV3 (Group III) is ^211^CTQQ^214^. The original sequence encoding the CaaX box may yet be unknown but one could envisage that any nucleotide variations leading to the coding of a varied CaaX box would be maintained as has, indeed, been observed in various HDV sequences.

Our previous findings have shown a greater extent of diversity in the sequence encoding for the CHC-interacting domain which could vary in sequences and locations [54]. The HDV1 (Group I) and HDV 2 and HDV4-8 (Group II) have a clathrin box sequence ^199^LFPAD^203^ and ^206^LPLLE^210^, respectively, while the HDV3 (Group III) does not have such a sequence (Table 1). Instead, HDV3 could still form a complex with CHC through the sequence ^205^YYWV^208^ or ^206^YWVP^209^ via the association with a CHC-adaptor protein, AP-2. Such binding flexibility could have further provided higher tolerance in nucleotide polymorphism in the HDV sequences.

## 6. Conclusion and Perspective

Interestingly, increasing lines of evidence show that the capsid protein or genome of many other RNA viruses binds to the same cellular factors as HDV does. For example, a pull-down assay of the capsid protein of mosquito- and blood-borne West Nile virus (WNV) has shown that the CHC is one of interacting proteins of the viral capsid protein [56]. Although the authors did not demonstrate that the interaction between the capsid protein of WNV and CHC is important for virion packaging, we could identify a consensus sequence of the clathrin box motif in the capsid proteins of all WNV isolates but not in the capsid proteins of Japanese encephalitis virus (JEV), a *Flavivirus* closely related to WNV (Table 2). Another example is the case of the HDV genome binding to glyceraldehydes 3-phosphate dehydrogenase (GAPDH), a key enzyme in glycolysis [57, 58]. The genomes of JEV and the tomato bushy stunt virus, a plant virus, are also found to bind to GAPDH [59, 60]. Whether the sequence of the HDV genome responsible for interaction with GAPDH also contributes to sequence diversity of the HDV genome awaits further investigation. 

As compared with many other satellite viruses which derived from a deletion of helper viruses, HDV lacks any similarity of nucleotide sequences of helper virus, the HBV, and evolved from two distinct nucleotide components through a recombination. In addition to discussing the possible origin of HDV, in this review, we proposed that the HDV nucleotide diversity could be resulted from the selection of interacting with host factors. By using the programs of PIST and PLATO, Anisimova and Yang analyzed all three codon positions of HDAg from 33 HDV isolates and explained that the HDV sequence diversity could be resulted from the positive selection force by escaping host immune response [61]. Nevertheless, it is a great challenge to bioinformatic scientists to formulate a set of general rules for the interpretation of the genome diversity in other RNA viruses.

## Figures and Tables

**Table 1:** Alignment of the nucleotide sequences that encode the carboxyl terminal 19-20 residues of LDAg. The sequences are displayed in three different groups (HDV1 as Group I, HDV2 and HDV4 to HDV8 as Group II, and HDV3 as Group III). The amino acid residues that constitute the clathrin box-binding domain and the isoprenylation signal are indicated above the nucleotide sequence in a single-letter amino acid abbreviation. The nucleotide substitution percentage is indicated at right, in which the total nucleotide variations within in-groups are indicated as “All” in the first column. The percentage of nucleotide variation corresponding to the clathrin-box binding domain and the isoprenylation site is shown in the second and third columns, respectively. The accession number of each HDV isolate is indicated at bottom.

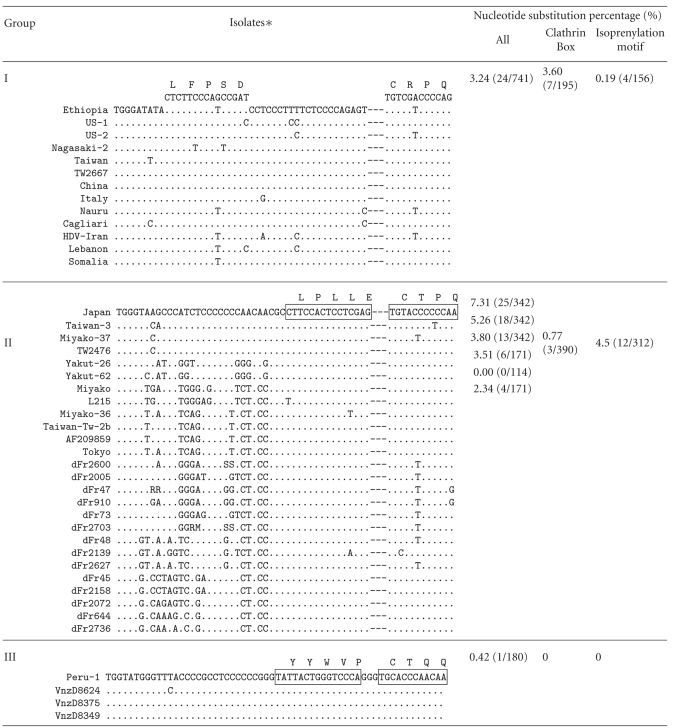

∗The accession numbers of the sequences used were **AF209859**, *AF209859*; **Cagliari**, *X85253*; **China**, *X77627*; **dFr45**, AX741144; **dFr47**, *AX741149*; **dFr48**, *AX741164*; **dFr73**, *AX741154;* **dFr644**, *AX741169*; **dFr910**, *AX741159*; **dFr2005**, *AM183331*; **dFr2072**, *AM183330*; **dFr2139**, *AM183332*; **dFr2158**, *AM183333*; **dFr2600**, *AM183326*; **dFr2627**, *AM183329*; **dFr2703**, *AM183328*; **dFr2736**, *AM183327*; **Ethiopia**, *U81989*; **HDV-Iran**, *AY633627*; **Italy**, *X04451*; **Japan**, *X60193*;** L215,** *AB088679*; **Lebanon**, *M84917*; **Miyako**, *AF309420*; **Miyako-36**, *AB118845*; **Miyako-37**, *AB118846*; **Nagasaki-2**, *AB118849*;** Nauru**, *M58629*; **Peru-1**, *L22063*; **Somalia**, *U81988*; **Taiwan**, *M92448*; **Taiwan-3**, *U19598*; **Taiwan-Tw-2b**, *AF018077*; **Tokyo**, *AB118847*; **TW2476**, *AF104264*; **TW266**7, *AF104263*; **US-1**, *D01075*; **US-2**, *L22066*; **Vnzd8349**, *AB037948*; **Vnzd837**5, *AB037947*; **Vnzd8624**, *AB037949*; **Yakut-26**, *AJ309879*; and **Yakut-62**, *AJ309880*.

**Table 2:** Alignment of the amino acid sequences of the capsid protein of the West Nile virus (WNV) with the Japanese encephalitis virus (JEV). Sequences derived from ten different isolates of WNV and JEV, respectively, were randomly selected from the public domain database for alignment. The GenBank accession number of each viral sequence is shown on the left. Red box shows the clathrin box present in the capsid of WNV but not in JEV.